# Comparison of Raw Acceleration from the GENEA and ActiGraph™ GT3X+ Activity Monitors

**DOI:** 10.3390/s131114754

**Published:** 2013-10-30

**Authors:** Dinesh John, Jeffer Sasaki, John Staudenmayer, Marianna Mavilia, Patty S. Freedson

**Affiliations:** 1 Health Sciences, Northeastern University, 316D Robinson Hall, 360 Huntington Ave., Boston, MA 02115, USA; 2 Department of Kinesiology, University of Massachusetts, Amherst, MA 01003, USA; E-Mails: Jesasaki@kin.umass.edu (J.S.); psf@kin.umass.edu (P.S.F.); 3 Department of Mathematics and Statistics, University of Massachusetts, Amherst, MA 01003, USA; E-Mail: jstauden@me.com; 4 College of Osteopathic Medicine, University of New England, ME 04103, USA; E-Mail: marimavilia@gmail.com

**Keywords:** wearable activity monitors, raw acceleration, physical activity

## Abstract

*Purpose*: To compare raw acceleration output of the ActiGraph™ GT3X+ and GENEA activity monitors. *Methods*: A GT3X+ and GENEA were oscillated in an orbital shaker at frequencies ranging from 0.7 to 4.0 Hz (ten 2-min trials/frequency) on a fixed radius of 5.08 cm. Additionally, 10 participants (age = 23.8 ± 5.4 years) wore the GT3X+ and GENEA on the dominant wrist and performed treadmill walking (2.0 and 3.5 mph) and running (5.5 and 7.5 mph) and simulated free-living activities (computer work, cleaning a room, vacuuming and throwing a ball) for 2-min each. A linear mixed model was used to compare the mean triaxial vector magnitude (VM) from the GT3X+ and GENEA at each oscillation frequency. For the human testing protocol, random forest machine-learning technique was used to develop two models using frequency domain (FD) and time domain (TD) features for each monitor. We compared activity type recognition accuracy between the GT3X+ and GENEA when the prediction model was fit using one monitor and then applied to the other. Z-statistics were used to compare the proportion of accurate predictions from the GT3X+ and GENEA for each model. *Results*: GENEA produced significantly higher (p < 0.05, 3.5 to 6.2%) mean VM than GT3X+ at all frequencies during shaker testing. Training the model using TD input features on the GENEA and applied to GT3X+ data yielded significantly lower (p < 0.05) prediction accuracy. Prediction accuracy was not compromised when interchangeably using FD models between monitors. *Conclusions*: It may be inappropriate to apply a model developed on the GENEA to predict activity type using GT3X+ data when input features are TD attributes of raw acceleration.

## 1. Introduction

In 2009, the American College of Sports Medicine and the National Institutes of Health co-sponsored the ‘Objective Measurement of Physical Activity: Best Practices and Future Directions’ conference to update best practice recommendations for using wearable monitors to assess physical activity [1]. A key conclusion of the conference was to limit the use of proprietary activity counts and utilize raw acceleration to estimate physical activity [1]. There are several advantages in using raw acceleration to estimate physical activity. The raw acceleration signal is a complex time-series waveform characterized by various features in multiple domains. For example, an acceleration signal has time-domain (TD) and frequency-domain (FD) features that are used as prediction variables to estimate attributes of physical activity and sedentary behavior [2,3]. Availability of numerous signal features greatly enhances the potential of using complex machine learning techniques to accurately estimate physical activity and sedentary behavior. These techniques are becoming increasingly popular, as they provide improved estimates as compared to the traditional activity count cut-points [3,4]. Another potential advantage of using raw acceleration is increased inter-monitor output equivalency through elimination of proprietary signal processing specifications used to derive activity counts. For example, activity counts from ActiGraph™ (ActiGraph™ Inc., Pensacola, FL, USA) monitors are not the same as those from the Actical (Phillips Respironics, Andover, MA, USA) monitor due to manufacturer specific signal processing [5]. While raw accelerometry is a possible solution for inter-monitor output equivalency, several sensor and digital signal processing specifications need to be similar between monitors to ensure equivalency.

Two activity monitors used in physical activity research are the ActiGraph™ GT3X+ and GENEA (Unilever Discover, Colworth, UK). These monitors have a dynamic range of ±6 g and allow users to collect raw acceleration at various sampling frequencies ranging from 10 to 160 Hz at 10 Hz increments. Currently, the GENEA is commercially unavailable, however, it is the only activity monitor that has been calibrated with an open-source machine-learning technique to predict the type of physical activity and sedentary behavior from raw acceleration [3]. Raw acceleration from the GT3X+ is currently being used in the National Health and Nutrition Examination Survey to obtain nationally representative physical activity and sedentary behavior estimates [6]. There is no evidence examining the equivalency of raw acceleration outputs from these monitors and whether an algorithm developed on one monitor can be applied to data from the other to produce similar activity type recognition accuracy. Thus, the purposes of this study were: (1) To compare mean vector magnitude, which is a computed metric of triaxial raw acceleration from both monitors during mechanical shaker testing at various oscillation frequencies and (2) to determine if there is an interaction-effect in predicting activity type when a prediction model developed on one monitor is applied to data from another monitor. We compare activity type recognition accuracy rates when a model developed using the GT3X+ is applied to GT3X+ and GENEA data, and *vice versa*.

## 2. Methods

### 2.1. Mechanical Shaker Testing

**Figure 1.**
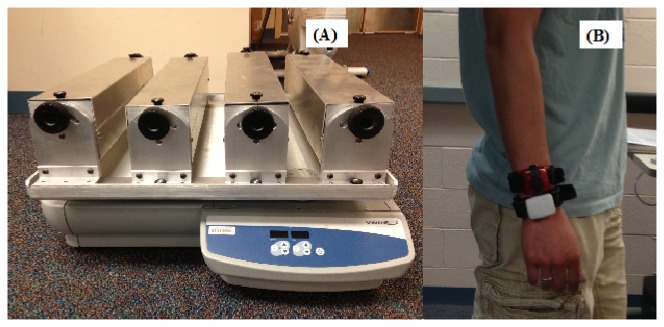
(**A**) Orbital mechanical shaker use for shaker testing, (**B**) Wrist worn GT3X+ and GENEA monitors.

Mechanical shaker testing was performed using an orbital shaker (VRW International, Radnor, PA, USA; Advanced Orbital Shaker, Model 10000-2) that produces controlled oscillations between 0.25 and 4.2 Hz. Oscillation radii can be adjusted between 1.27 and 5.7 cm. Four trays (51 × 10 × 10 cm) were mounted on the base oscillating plate of the shaker. Each tray had five slots to securely hold activity monitors in place and eliminate movement during orbital shaking. Prior to data collection, we performed shaker testing using 4 GT3X+ and GENEA monitors to establish inter-unit reliability for each monitor type. Intra-monitor coefficient of variation was less than 1.6% for both monitors. This is similar to previous reports of intra-monitor reliability for ActiGraph™ and GENEA monitors using MEMS capacitive sensors [7,8]. A single GT3X+ and GENEA were initialized to collect data at a sampling frequency of 80 Hz and were oscillated during 10 trials. Each trial lasted 10 min (five frequencies × 2 min each) and consisted of monitor oscillation at 0.7, 1.3, 2.3, 3.3 and 4.0 Hz on a fixed radius of 5.08 cm [9,10]. These frequencies are similar to those observed during ambulation at speeds ranging between 1.5 to 16 mph [11]. The activity monitors were randomly positioned to a different slot prior to each trial and no device was oscillated in the same slot more than one time. Figure 1A depicts the mechanical shaker used in the study.

### 2.2. Human Testing

Eight participants (mean ± SD: age = 23.8 ± 5.4 years; Body Mass Index = 22.7 ± 1.4 kg·m^2^) were recruited from the University of Massachusetts, Amherst and the surrounding community. The University of Massachusetts, Amherst Institutional Review Board, approved the experimental protocol and all participants provided written informed consent. Participants visited the Physical Activity and Health Laboratory to perform the human testing protocol. Participants wore activity monitors at the wrist on two Velcro^®^ wristbands while performing the activity protocol. The monitors were positioned such that one was distal to the other when the arm was straight and pointing downwards on the side of the body. We minimized residual confounding due to placement effect by counterbalancing proximal/distal monitor placement. Figure 1B illustrates monitor placement on the wrist.

The lab-testing protocol included treadmill and simulated free-living activities. Participants walked at 2.0 and 3.5 mph and ran at 5.5 and 7.5 mph on a treadmill for 2 min each. These were followed by 2 min of seated computer-work vacuuming, cleaning the room and throwing a ball. The activities were selected to cover a wide range of dynamic acceleration between 0 and 6 g. Start and stop times for all activities were recorded.

### 2.3. Data Analyses

Inter-monitor comparisons during shaker testing used data from the 2nd minute of each two-minute trial. Linear mixed models with likelihood ratio tests (p < 0.05) were used to compare mean triaxial vector magnitude of raw acceleration between the GT3X+ and GENEA at each oscillation frequency. We used a Random Forest [12] to develop activity type recognition models using the GT3X+ and GENEA. Two prediction models were developed for each monitor. One model primarily used frequency domain (FD) features as predictor variables and the other used time domain features. We assessed the performance of models using FD and TD features because these two types are most commonly used as machine learning input features to estimate physical activity. The input features for the FD models were mean acceleration, total signal power, frequency of the signal with most power, power in 0.6 to 2.5 Hz, power in 0.6 to 2.5 Hz divided by total power and the dominant frequency at the 10th and 90th percentiles of the power spectral density. The input features for the TD models were the mean, standard deviation, 10th, 25th, 50th, 75th and 90th percentiles of signal distribution and lag-1-autocorrelation of the acceleration signal. Features for FD and TD models were extracted from 20-second intervals of data from the last minute of each activity. Thus, 24 samples for each activity were used to train and test the prediction models. We determined prediction accuracy for each type of model when the development and testing data were from the same monitor (*i.e.*, GT3X+ model on GT3X+ data, GENEA model on GENEA data) and when the development and testing data were from different monitors (GT3X+ model on GENEA data, GENEA model on GT3X+ data). These comparisons were made using Z-statistics (p < 0.05) and all results were cross-validated using leave-one out analyses.

## 3. Results

**Figure 2.**
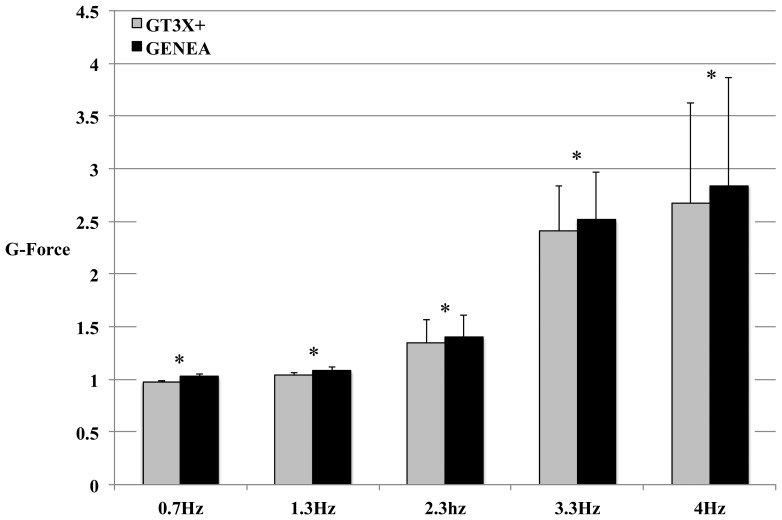
Comparison of the mean triaxial raw acceleration vector magnitude at four different oscillation frequencies during mechanical shaker testing. (* indicates statistically significant differences, p < 0.05. Error bars represent standard deviations).

**Figure 3.**
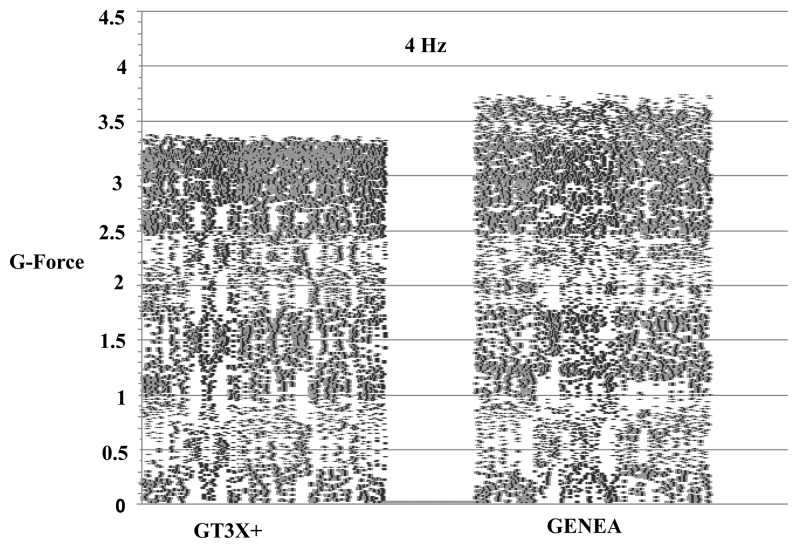
Comparison of absolute raw acceleration between the GT3X+ and GENEA at 4 Hz for the x-axis during one trial of shaker testing.

Raw acceleration vector magnitudes were significantly different between the GT3X+ and the GENEA during shaker testing at all four oscillation frequencies. Figure 2 shows a comparison of the mean triaxial vector magnitude of raw accelerations during mechanical shaker testing at all five oscillation frequencies in the x-axis. The differences ranged between 3.5 to 6.5% and increased with increasing oscillation frequency. Figure 3 compares absolute raw acceleration for one minute at the highest frequency of 4 Hz detected by the x-axis of both monitors during one trial of shaker testing. The x-axis represents the vertical axis when the devices are worn on the hip. The largest observed inter-monitor difference in peak raw acceleration was approximately 7.4% at a frequency of 4 Hz. This magnitude of difference was also observed in the y-axis, which was sensitive to the oscillatory motion of the mechanical shaker. Both axes were not sensitive to the force of gravity.

Overall activity type recognition accuracy using the 4 models developed on GT3X+ and GENEA data are presented in Table 1. There were no significant differences in overall prediction accuracy when models developed using FD features were used on data from the same monitor or on data from the other monitor. In other words, an FD model developed on GT3X+ data produced similar activity type recognition accuracy when applied to GT3X+ data or GENEA data.

Similar results were observed for a prediction model developed using GENEA data. There was no statistically significant difference (p < 0.05) in activity type recognition accuracy when the TD feature model developed and tested on GT3X+ data was compared to recognition accuracy when the GT3X+ model was tested on GENEA data. However, there was a significant difference (p < 0.05) in activity type recognition accuracy when the TD feature model developed and tested on GENEA data was compared to prediction accuracy when the same model was tested on GT3X+ data. Table 2 is activity type classification confusion matrices when the TD feature model developed on GENEA data was tested on GENEA data and when the same model was tested on GT3X+ data.

## 4. Discussion

We examined if VM of triaxial raw acceleration signals from the GT3X+ and GENEA were similar to each other during mechanical shaker testing and if an activity type recognition model developed using data from one activity monitor could be applied to that from the other and yield similar prediction accuracy.

### 4.1. Shaker Testing

The acceleration response of the GENEA during mechanical shaker testing was consistently higher than the GT3X+. The difference in response between monitors increased as the magnitude of acceleration increased (Figure 2). We computed centrifugal acceleration for each oscillation frequency to determine which monitor produced a more accurate acceleration response. Table 3 compares computed centrifugal acceleration at each oscillation frequency to average peak acceleration from the GT3X+ and GENEA detected in the x-axis. Centrifugal acceleration was calculated as ‘*a* = ω^2^*r*’*,* where ‘*a*’ is centrifugal acceleration, ω is angular velocity and ‘*r*’ is radius of oscillation. Average peak acceleration from the monitors is computed from the two acceleration peaks on either side of baseline (0 g) in every revolution for one minute. Peak acceleration response from the capacitive accelerometers in the GT3X+ and GENEA most closely reflect the constant centrifugal acceleration detected during orbital shaker testing. The GT3X+ produced acceleration values that were closer to the computed centrifugal acceleration during all frequencies. The mechanical shaker in this study had a maximum oscillation capacity of 4.2 Hz, which subject the monitors to a maximum acceleration of around 3.5 g. Thus, we were unable to examine the magnitude of difference in acceleration between monitors at their highest dynamic capacity (±6 g). Based on current findings, it seems that the inter-monitor differences would increase beyond 0.4 to 0.5 g at oscillation frequencies that generate acceleration closer to ±6 g.

### 4.2. Human Testing

There was minor but non-significant degradation (less than 3%) in activity type accuracy for the FD models when predictions were made using data from the other monitor (Table 1). When the TD activity type recognition model developed on the GT3X+ was applied to GENEA data, prediction accuracy was 2% lower in comparison to when the model was tested on GT3X+ data. In contrast, using the TD activity type recognition model developed on the GENEA on GT3X+ data yielded a relatively large reduction in accuracy (∼) 8% when compared to results when testing the model on GENEA data. Decline in accuracy was mainly due to a high rate of misclassifying cleaning the room as walking. The former is an intermittent activity while walking is a highly rhythmic activity. A closer examination revealed that cleaning the room was mostly misclassified as walking at a slow speed of 2.0 mph. Similarly, a more rhythmic activity like vacuuming (repetitious hand movements) was misclassified as cleaning the room. The reduction in recognition accuracy for these two misclassifications was approximately 40 and 11%, respectively. This may be because TD features typically are representations of actual g-force measures, which may be the same for two different activity types. It is possible that the inter-monitor discrepancies caused the acceleration of the wrist detected by the GENEA during cleaning the room and vacuuming to be similar to that during slow walking and cleaning the room as detected by the GT3X+. This misclassification may be problematic if the goal is to distinguish between activity behavior types, e.g., ambulatory steps *vs.* household activities.

Misclassification of cleaning the room and vacuuming may have been attenuated when using the FD model because FD features are characteristics of the frequency spectrum that can account for rhythmicity. Another reason for similar prediction accuracy when interchangeably using the FD model between the GT3X+ and the GENEA may be because inter-monitor differences in the FD features are minimal. Patterns of the raw signal from both devices may be similar to each other even though the magnitude of the signal from the GENA is greater than that from the GT3X+. For instance, although the GENEA may produce a higher acceleration response of 1.7 g while walking at 3.5 mph as compared to 1.5 g by the GT3X+, the difference of 0.2 g may minimally affect the dominant frequency of the acceleration signal (1.5 Hz) from both devices. We statistically compared the features used in the two types of models for a monitor effect using linear models and likelihood ratio tests. We found significant inter-monitor differences for all TD features (p < 0.05). For the 6 FD features, we found significant inter-monitor differences (p < 0.05) only for total power and frequency below 90% of total power. However, these differences were small in magnitude and may have been insufficient to cause significant differences in activity type recognition when the FD model developed on one monitor was applied to data collected from the other monitor.

### 4.3. Signal Processing Factors

Some factors that may cause inter-monitor differences in raw acceleration are related to signal processing and digitization of the analog signal output by the MEMS sensor in an activity monitor. A possible factor is the low pass anti-aliasing filtering of the analog signal from the MEMS sensor. This filtering is performed to minimize signal distortion during analog to digital conversion. The GT3X+ utilizes the Kionix^®^ (Ithaca, NY, USA) KXSC7-3672 accelerometer and the sensor in the GENEA is the LIS3LV02DL (ST Microelectronics, Geneva, Switzerland). Both sensor manufacturers offer preset and user-programmable low-pass filtering. The GENEA sensor has programmable filters at 50 Hz increments. Conversely the GT3X+ sensor has pre-selectable filters of 2 kHz, 1 kHz, 500 Hz, 100 Hz, 50 Hz, or no low pass filter. However, monitor manufacturers can define their own low-pass filter with external capacitors. ActiGraph™ adopts this strategy and these filter specifications are proprietary [11]. These inter-monitor differences in low-pass filtering of analog signals may result in differences in raw acceleration. Some other factors are differences in the zero-g offset, reference voltage, analog-to-digital bit-rate conversion, and sensitivity of the MEMS acceleration sensor [13].

## 5. Conclusions

We found significant differences between the GT3X+ and the GENEA triaxial VM of raw accelerations during shaker testing. These differences will affect the applicability of a random forest machine learning method using TD features developed on one monitor to predict activity type using raw acceleration VM data from another. However, this was not the case when using a random forest model that uses FD features. These findings may be applicable when using absolute raw acceleration or other metrics. Thus, it may be advisable to avoid using raw acceleration TD features as predictors when developing a model especially if the model is intended to be used with data from another monitor. However, there may be several research questions that require the measurement and use of TD features to determine study outcomes. An example is the study of impact forces and its relation to bone development. In these cases, it may be advisable to avoid the interchanging of prediction models between different activity monitors. Primarily using FD features in a prediction model may greatly increase monitor equivalency, especially when predicting categories of physical activity attributes like gross activity type (e.g., ambulation, lifestyle) and activity intensity categories (e.g., sedentary, light, moderate).

A limitation of our study was a small sample size for the human testing protocol (24 samples). However, our findings may inform the research community that it may be unrealistic to expect complete inter-monitor output equivalency when using commercially available activity monitors. A strength of the study is that we examined inter-monitor differences using shaker testing and demonstrated the practical implications of these findings during human testing. Future research needs to determine if a correction factor can be applied to increase monitor output equivalency between the GT3X+ and the GENEA. Given the pattern of increasing differences with increasing g-force, a correction factor may entail simple exponential scaling of output from one monitor to match the other. However, the ideal correction factor would require complete transparency of all proprietary signal filtering specifications that can be used to minimize inter-monitor differences. Research must also examine if it is possible to extensively train machine learning algorithms to better utilize and recognize physical activity attributes so that models become interchangeable and can be used in different activity monitors.

## 6. Disclosure

Dr. Patty Freedson is a member of ActiGraph Scientific Advisory Board.

## Figures and Tables

**Table 1.** Overall activity type classification accuracy when a model developed on data from one monitor is applied to data from the same monitor and the other monitor. (AG = GT3X+, GE = GENEA, FD = Frequency Domain, TD = Time Domain). (* indicates statistically significant differences, p < 0.05.).

**Model**	**Training Monitor**	**Prediction Monitor**	**Prediction Accuracy (%)**
FD	AG	AG	95.8
	AG	GE	93.8
	GE	GE	94.3
	GE	AG	93.2

TD	AG	AG	91.7
	AG	GE	89.6
	GE	GE	94.3
	GE	AG	86.5

**Table 2.** Activity type classification accuracy using the TD feature model. (**A**) Trained on GENEA and predicted using GENEA, (**B**) Trained on GENEA and predicted using GT3X+.

	**CLEAN**	**COMP**	**THROW**	**WALK**	**RUN**	**VAC**	**% correct**
**CLEAN**	**19**	0	0	5	0	0	**79.2%**
**COMP**	0	**23**	1	0	0	0	**95.8%**
**THROW**	0	2	**18**	2	0	0	**81.8%**
**WALK**	1	0	0	**47**	0	0	**97.9%**
**RUN**	0	0	0	0	**48**	0	**100.0%**
**VAC**	0	0	0	0	0	**24**	**100.0%**
							**total % correct 94.3%**

(**A**)							

	**CLEAN**	**COMP**	**THROW**	**WALK**	**RUN**	**VAC**	**% correct**
**CLEAN**	**9**	0	0	15	0	0	**37.5%**
**COMP**	0	**19**	5	0	0	0	**79.2%**
**THROW**	0	0	**21**	1	0	0	**95.5%**
**WALK**	0	0	0	**48**	0	0	**100.0%**
**RUN**	0	0	0	0	**48**	0	**100.0%**
**VAC**	4	1	0	0	0	**19**	**79.2%**
							**total % correct 86.5%**

(**B**)							

**Table 3.** Comparison of computed centrifugal acceleration and mean peak accelerations from the GT3X+ and GENEA monitors (x-axis) in g-force.

**Hz**	**Centrifugal Accn. (G)**	**GT3X+ (G)**	**GENEA (G)**
0	NA	0.007	0.006
0.7	0.091	0.123	0.169
1.3	0.363	0.397	0.435
2.3	1.113	1.155	1.192
3.3	2.272	2.301	2.395
4.0	3.271	3.241	3.483

## References

[1] Freedson P., Bowles H.R., Troiano R., Haskell W. (2012). Assessment of physical activity using wearable monitors: recommendations for monitor calibration and use in the field. Med. Sci. Sport Exerc..

[2] Staudenmayer J., Pober D., Crouter S., Bassett D., Freedson P. (2009). An artificial neural network to estimate physical activity energy expenditure and identify physical activity type from an accelerometer. J. appl. Physiol..

[3] Zhang S., Rowlands A.V., Murray P., Hurst T.L. (2012). Physical activity classification using the GENEA wrist-worn accelerometer. Med. Sci. Sport Exerc..

[4] John D., Liu S., Sasaki J.E., Howe C.A., Staudenmayer J., Gao R.X., Freedson P.S. (2011). Calibrating a novel multi-sensor physical activity measurement system. Physiol. Meas..

[5] John D., Freedson P. (2012). ActiGraph and Actical physical activity monitors: A peek under the hood. Med. Sci. Sport Exerc..

[6] National Health and Nutrition Examination Survey: Physical Activity Monitor (PAM) Procedures Manual. http://www.cdc.gov/nchs/data/nhanes/nhanes_11_12/Physical_Activity_Monitor_Manual.pdf.

[7] Esliger D.W., Rowlands A.V., Hurst T.L., Catt M., Murray P., Eston R.G. (2011). Validation of the GENEA Accelerometer. Med. Sci. Sport Exerc..

[8] Santos-Lozano A., Marin P.J., Torres-Luque G., Ruiz J.R., Lucia A., Garatachea N. (2012). Technical variability of the GT3X accelerometer. Med. Eng. Phys..

[9] Orendurff M.S., Segal A.D., Klute G.K., Berge J.S., Rohr E.S., Kadel N.J. (2004). The effect of walking speed on center of mass displacement. J. Rehabil. Res. Dev..

[10] Rothney M.P., Apker G.A., Song Y., Chen K.Y. (2008). Comparing the performance of three generations of ActiGraph accelerometers. J. Appl. Physiol..

[11] John D., Miller R., Kozey-Keadle S., Caldwell G.C., Freedson P.S. (2012). Biomechanical examination of the ‘plateau phenomenon’ in ActiGraph vertical activity counts. Physiol. Meas..

[12] John D., Staudenmayer J., Freedson P. (2013). Simple to complex modeling of breathing volume using a motion sensor. Sci. Total Environ..

[13] Miller J. Accelerometer Technologies, Specifications and Limitations.

